# Sphenoid Sinus Aspergilloma in an Immunocompetent and an Immunocompromised Patient: A Case Report

**DOI:** 10.7759/cureus.34517

**Published:** 2023-02-01

**Authors:** Manraj Singh, Brittany M Zaita, Deepinder Singh, Adityabikram Singh, Gurjinder Kaur

**Affiliations:** 1 Department of Basic Biomedical Sciences, Dayanand Medical College and Hospital, Ludhiana, IND; 2 Department of Basic Biomedical Sciences, Touro College of Osteopathic Medicine, Middletown, USA; 3 Department of Neurosurgery, Satguru Partap Singh (S.P.S. Hospital, Ludhiana, IND; 4 Department of Basic Biomedical Sciences, Rutgers University New Jersey Medical School, Newark, USA; 5 Department of Physiology, Touro College of Osteopathic Medicine, Middletown, USA

**Keywords:** immunocompetent, immunocompromised, transsphenoidal surgical resection, voriconazole, sellar mass, invasive aspergillosis, cns aspergilloma

## Abstract

Sellar, supra-sellar aspergilloma are rare differentials for a sellar mass. CNS aspergilloma occurs due to intracranial extension of invasive fungal sinusitis, and often first manifests with symptoms of headache and visual disturbance. This complication is much more common in immunocompromised patients, but proliferation of fungal pathogens and low index for suspicion has led to more severe breakthrough cases in the immunocompetent. If treated timely, these CNS lesions can have a relatively good prognosis. Conversely, delays in diagnosis can confer very high rates of mortality among patients with invasive fungal disease. Originally from India, in this case report, we describe two patients presenting with sellar, supra-sellar tumors, who eventually were diagnosed with confirmed cases of invasive intracranial aspergilloma. We describe the clinical presentation, imaging techniques, and treatment modalities for this relatively rare disease in both the immunocompromised and the immunocompetent.

## Introduction

Invasive aspergillosis (IA) is a rare cause of mortality in immunocompetent patients, however, in recent years it seems there should be an increased focus on understanding more about these sporadic cases in previously healthy individuals. Conversely, IA is a relatively common cause of mortality in patients with prior hematologic malignancy or solid-organ transplant. *Aspergillus* is a species of ubiquitous mold found in soil; *A. fumigatus* is the most common pathogen causing IA but other organisms within the species, such as *A. flavus*, *A. terreus*, and *A. niger* can also lead to rare, treatment-resistant forms of the disease [1]. Classically documented high-risk factors for the development of IA include severe neutropenia, high-dose glucocorticoid use, immunosuppressive diseases or drug regimens, or a history of transplants [2]. This list may be expanded to notably include chronic diseases such as liver failure, COPD, TB, and diabetes, which also weaken immune barrier defenses and lead to increased susceptibility to disseminated infection [1].

Documentation of sporadic cases of IA, including those in the immunocompetent population, are mainly from China, India, and Thailand. It is thought that *Aspergillus* species are more pervasive in humid tropical and subtropical regions [1]. The case series presented in this report include patients who were diagnosed and treated in India. Though aspergillosis is considered primarily a pulmonary disease; where inhalation exposures allow the pathogen to directly colonize the paranasal sinuses, immunocompromised hosts have an increased propensity for hematogenous spread involving the skin, brain, eyes, liver, or kidneys [2].

CNS aspergilloma is a mass lesion that occurs due to invasive intracranial extension of fungal sphenoid sinusitis. This aggressive form of the disease involves critical structures such as the cavernous sinus, pituitary gland, and cranial nerves II, III, IV, V1, V2, and VI. Common presenting features of early fungal sphenoid sinusitis include headache (61.8%), visual disturbances (57.4%), and facial/orbital pain (30.9%). CNS aspergilloma is extremely rare in patients who lack significant risk factors or medical history. Additionally, intracranial aspergillosis is often misdiagnosed due to the limitations of distinguishing the mass on radiologic imaging alone. These pathologies can mimic sella, suprasellar or clival tumors, meningioma, trigeminal schwannoma, and inflammation of paranasal air sinuses. Intracranial aspergillosis carries a mortality rate as high as 80%, therefore early detection and management with adequate surgical intervention and antifungal therapy are crucial to overall patient prognosis [3].

## Case presentation

Patient 1

**Figure 1:**
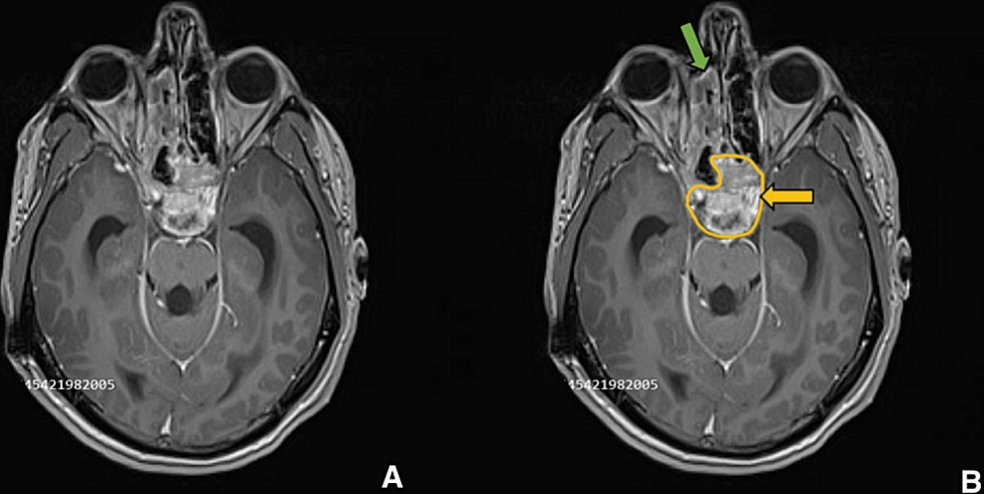
Preoperative imaging for patient 1. Contrast-enhanced T1-weighted axial image showing sellar, suprasellar lesion centered over clivus, measuring 4.5 x 5.1 x 3.1 cm. (A) Without ROI, and (B) with ROI. The yellow arrow points to the lesion which extends anteriorly to invade the sphenoid sinus. The green arrow shows the involvement of the right ethmoid sinus.

**Figure 2:**
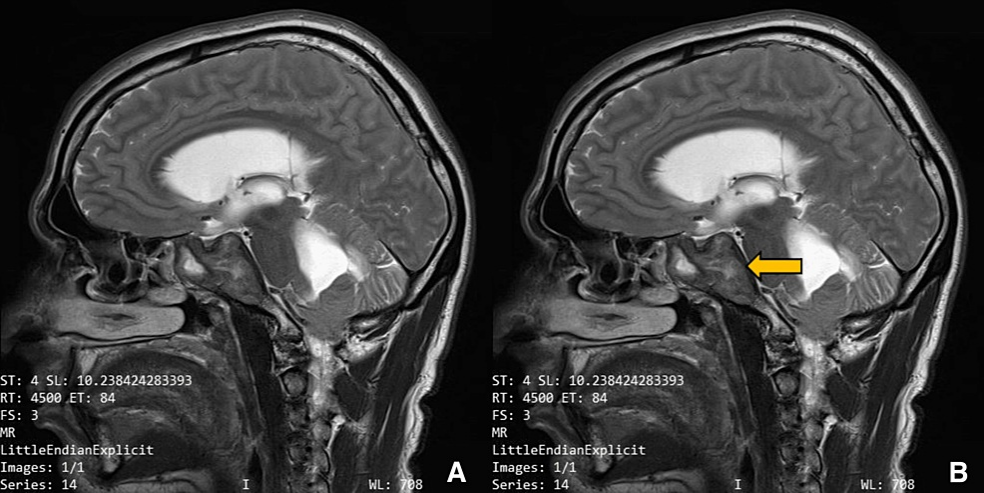
Preoperative imaging for patient 1. Plain T2-weighted sagittal image showing sellar, suprasellar lesion. (A) Without ROI, and (B) with ROI. The yellow arrow points to the lesion which is hypointense surrounded by hyperintense regions which are specific to *Aspergillus* infection.

**Figure 3:**
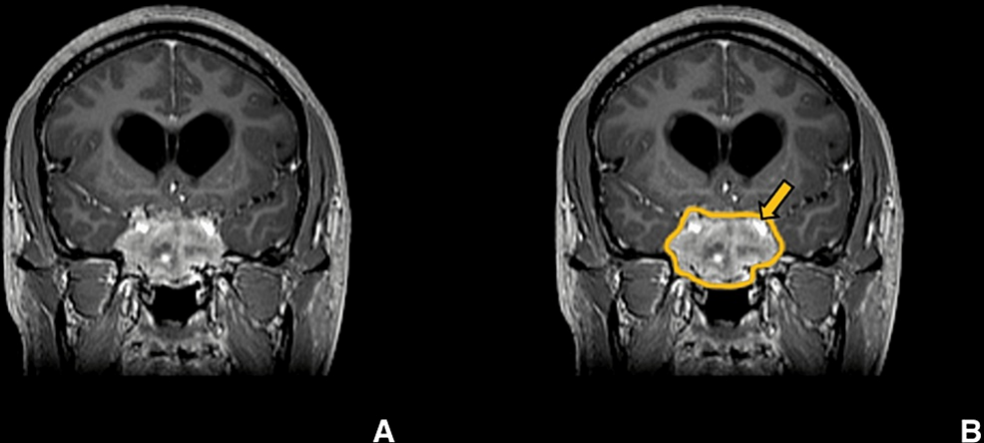
Preoperative imaging for patient 1. Contrast-enhanced T1-weighted coronal image showing sellar, suprasellar lesion with cavernous sinus invasion, which was more pronounced on the right side. (A) Without ROI, and (B) with ROI.

**Figure 4:**
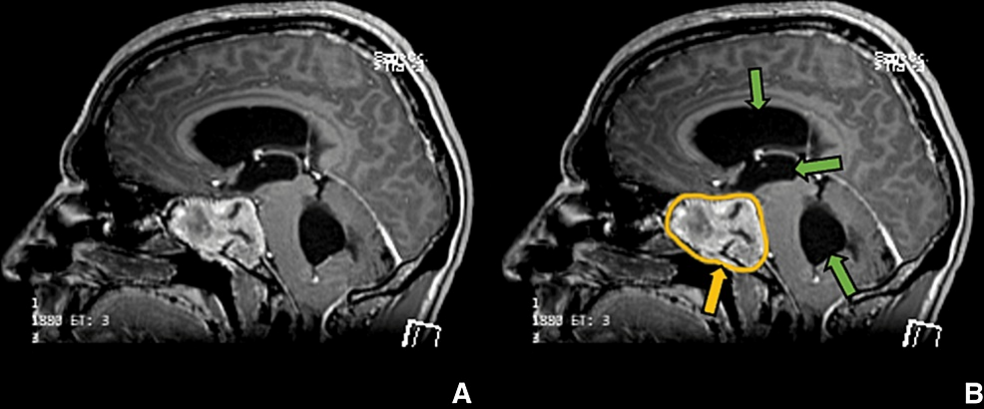
Preoperative imaging for patient 1. Contrast-enhanced T1-weighted sagittal image showing sellar, suprasellar lesion involving clivus and sphenoid sinus. (A) Without ROI and (B) with ROI. The green arrows show hydrocephalus of the lateral, third, and fourth ventricles.

**Figure 5:**
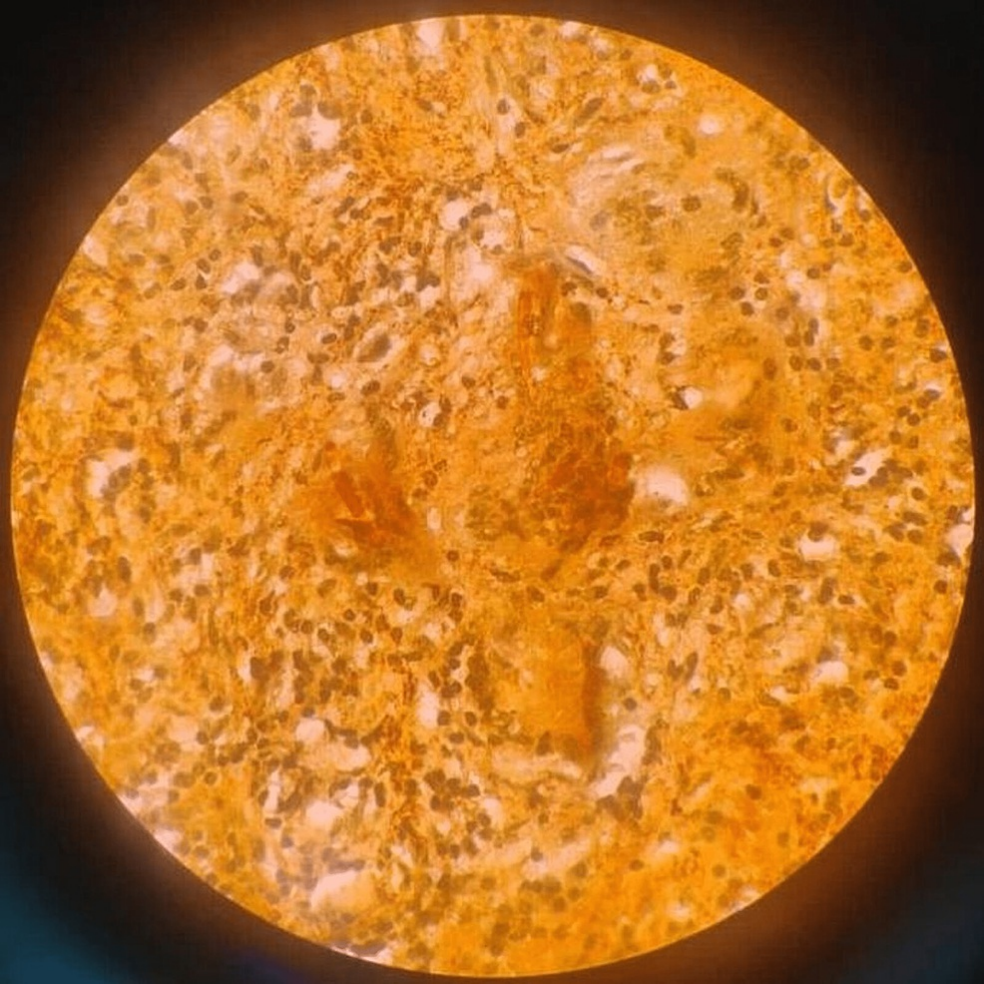
Pathology specimen image for patient 1. Dense infiltration of plasma cells, lymphocytes, neutrophils, and eosinophils. Epithelioid histocytes forming a granuloma.

**Figure 6:**
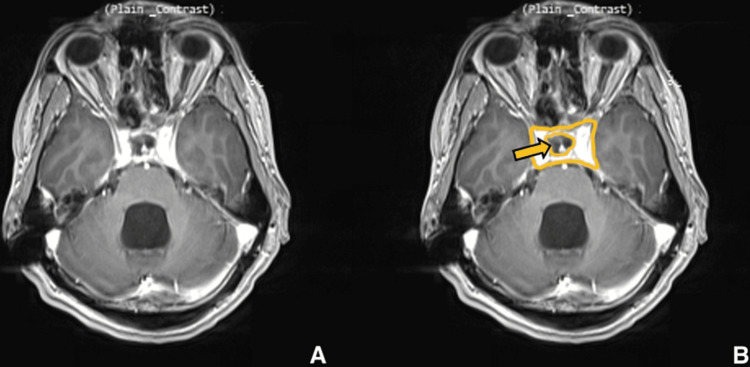
Postoperative imaging for patient 1. Contrast-enhanced T1-weighted axial image showing significant reduction in size of lesion. (A) Without ROI and (B) with ROI. The yellow arrow is pointing to the hypointense region where the mass has been reduced.

**Figure 7:**
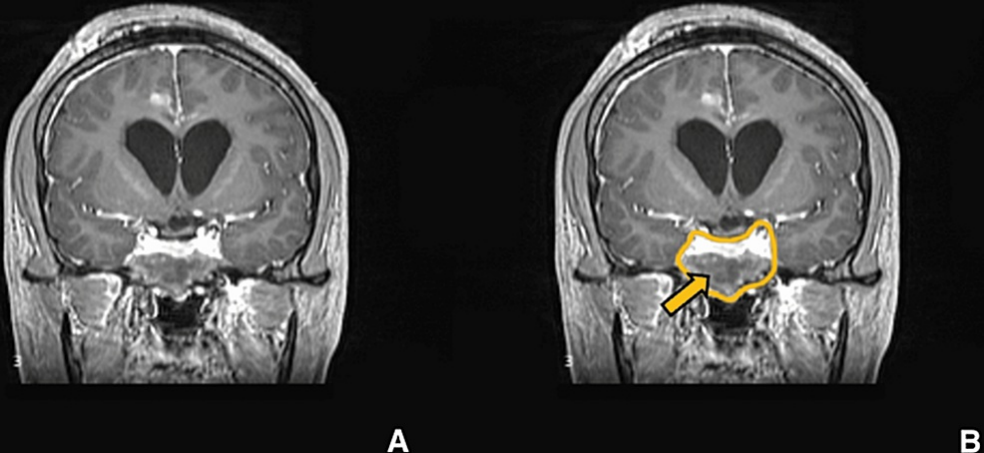
Postoperative imaging for patient 1. Contrast-enhanced T1-weighted coronal image showing significant reduction in size of the lesion. (A) Without ROI and (B) with ROI. The yellow arrow is pointing to the hypointense region where the mass has been reduced.

**Figure 8:**
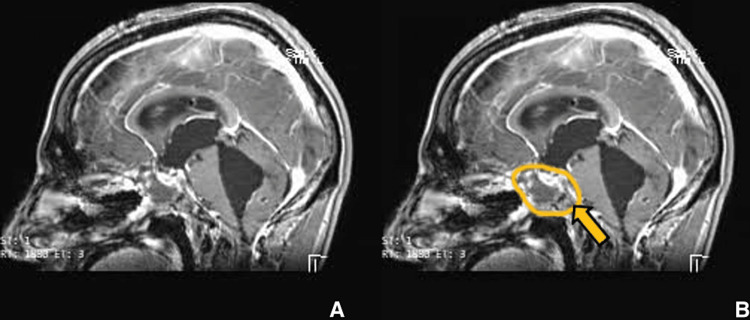
Postoperative imaging for patient 1. Contrast-enhanced T1-weighted sagittal image showing significant reduction in lesion size. (A) Without ROI and (B) with ROI. Post-surgical changes are shown, including interventional ventriculoperitoneal (VP) shunt.

Our patient is a 28-year-old male with no known medical history who presented with complaints of severe headache and vomiting over the last month. On arrival, the patient was conscious, oriented, and afebrile. A neurological examination revealed bilateral papilledema and bitemporal field cuts with decreased visual acuity. MRI showed a large mass measuring 4.5 x 5.1 x 3.1 cm. The mass was isointense on T1-weighted images (Figures 1A, 1B) and hyperintense on T2-weighted images (Figures 2A, 2B) with heterogeneous enhancement with IV contrast. The mass was primarily invading the sphenoid sinus, ethmoid sinus, sella, and clivus. The pituitary gland was seemingly displaced superiorly per the initial radiology report; however, T2WI shows the pituitary and lesion cannot be well differentiated. The mass was invading the prepontine cistern, abutting the basilar artery, and laterally the mass was invading the right cavernous sinus and encasing the right carotid artery (Figures 3A, 3B). There was also evidence of hydrocephalus with enlarged lateral, third, and fourth ventricles along with periventricular ooze (Figures 4A, 4B). His hormonal profile was normal. The patient was taken up for surgery with a working diagnosis of invasive pituitary adenoma vs. chordoma vs. sarcoma. Following VP shunt for symptomatic hydrocephalus, he underwent subtotal endoscopic decompression of a very hard tumor which was relatively avascular. Histopathology revealed fibrocollagenous tissue with moderate to dense infiltration with plasma cells, lymphocytes, eosinophils, and neutrophils. There was a focal collection of epithelioid histiocytes forming a granuloma. Few septate, branched fungal hyphae morphologically suggestive of *Aspergillus* species were seen (Figures 5A, 5B). Antifungal treatment with voriconazole (400mg/day) was initiated at this time. Follow-up MRI at six months showed significant resolution of spheno-sellar-suprasellar fungal mass as well as an improvement of clinical symptoms (Figures 6-8). The patient is still currently under follow-up and on 400mg/day of voriconazole.

Laboratory testing was performed to rule out other comorbid medical conditions which showed that anti-HCV, HBsAg, and HIV 1 and 2 antibodies were non-reactive. The patient was screened for diabetes, and a random, non-fasting blood glucose was 130 mg/dL, therefore further screening such as HbA1C was not done at this time. CSF studies were within normal limits. A CBC showed an elevated PCV and MCHC and a decreased MCV. A mild microcytic anemia may be indicated in this patient due to what we understand about *Aspergillus* and consumption of iron. The patient also had a mildly elevated PT but did not experience any major hemorrhage or post-surgical complications. Other notable laboratory values for this patient included a serum alkaline phosphatase which was elevated to 170 U/L with otherwise normal LFTs. The patient also exhibited a very mildly elevated serum globulin (3.7 gm/dL) and subsequently a mildly decreased A/G ratio (1.16), likely a sign of the invasive disease. The remainder of the laboratory values obtained were within normal limits and are further detailed in Table 1. A chest x-ray was performed which showed bilateral increased bronchovascular markings. Additionally, a SARS-CoV-2 PCR was negative at the time of admission. A further detailed medical history, including childhood history and vaccination status, was unable to be obtained for this patient.

**Table 1:** Laboratory studies from patient 1

Test	Result	Reference Range
SARS-CoV-2 RNA RT PCR	Negative	Negative
Anti-HCV (rapid)	Non-Reactive	—
HbsAg (rapid)	Non-Reactive	—
HIV (rapid)	Non-Reactive	—
Random blood glucose (non-fasting)	130	<140 mg/dL
CSF Studies		
CSF description	5 mL, clear, pale yellow	—
CSF Glucose	66	40-70 mg/dL
CSF Protein	17.3	15-45 mg/dL
Complete Blood Count		
Hemoglobin	15.3	13-17 g/dL
Total Leukocyte Count	8.1	4-10 x 10^3^/uL
RBC Count	5.35	4.5-5.5 x 10^6^/uL
ESR	14	mm/hr
Packed Cell Volume	43.6	40-42%
Mean Corpuscular Volume	81.5	83-101 fl
Mean Corpuscular Hemoglobin	28.7	27-32 pg
MCHC	35.2	31.5-34.5 g/dL
RDW	13.4	11-16%
Platelet	2.11	1.5-4 Lakhs/cumm
MPV	8.6	fl
Neutrophils	65	40-80%
Lymphocytes	28	20-40%
Monocytes	5	2-10%
Eosinophils	2	1-6%
PT/PT-INR		
PT	15.8	11-15 seconds
Control	13.3	seconds
INR	1.18	seconds
Liver Function Test/Profile-serum		
Total Bilirubin	0.2 mg/dL	0.2-1 mg/dL
Direct Bilirubin	0.1 mg/dL	0-0.2 mg/dL
Indirect Bilirubin	0.1 mg/dL	0-1 mg/dL
Total Protein	8 g/dL	6.3-8.2 g/dL
Albumin	4.3 g/dL	3.5-5 g/dL
Globulin	3.7 g/dL	2-3.5 g/dL
A/G Ratio serum	1.16	1.3-2
SGOT/AST	14 U/L	5-40 U/L
SGPT/ALT	34 U/L	5-40 U/L
Alkaline Phosphatase	170 U/L	46-116 U/L
GGT-serum	54 U/L	5-85 U/L
Renal Profile-serum		
Urea	32 mg/dL	15-39 mg/dL
Creatinine-serum	0.72 mg/dL	0.5-1.3 mg/dL
Uric Acid	3.6 mg/dL	2.5-7.5 mg/dL
Sodium (Na^+^) serum	140 mEq/L	135-145 mEq/L
Potassium (K^+^) serum	4.3 mEq/L	3.5-5.1 mEq/L
Chloride (Cl^-^) serum	105 mEq/L	98-107 mEq/L
Calcium serum	9.3 mg/dL	8.5-10.1 mg/dL
Phosphorus serum	4.1 mg/dL	2.5-4.5 mg/dL

Patient 2

Our patient is a 26-year-old HIV-positive immunocompromised female who presented with complaints of headache, vomiting, and altered sensorium for the last 15 days. On initial presentation, the patient was drowsy with GCS of E3V4M6. Neurological examination revealed a significant visual loss in the form of light perception only. Prior to the presentation, this patient was on an antiretroviral regimen of Doultegavir + Lamivudine + Tenofovir disoproxil fumarate 1 tablet once daily. No prophylactic medications for this patient were known. An HIV viral load was not able to be obtained for this patient.

**Figure 9:**
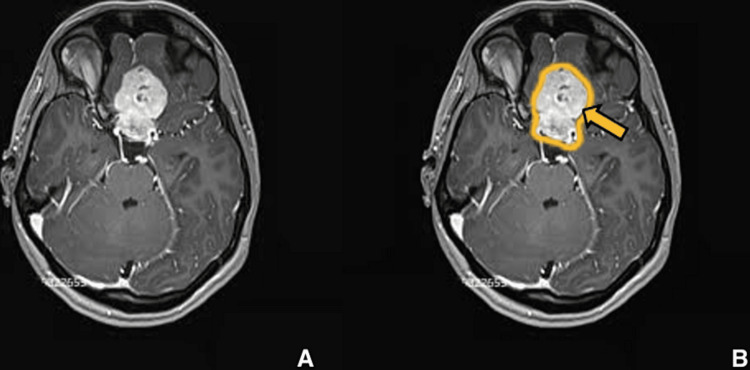
Preoperative imaging for patient 2. Contrast-enhanced T1-weighted axial image showing lesion involving sellar, suprasellar region measuring 3.6 x 4.7 x 8.5 cm. (A) Without ROI and (B) with ROI.

**Figure 10:**
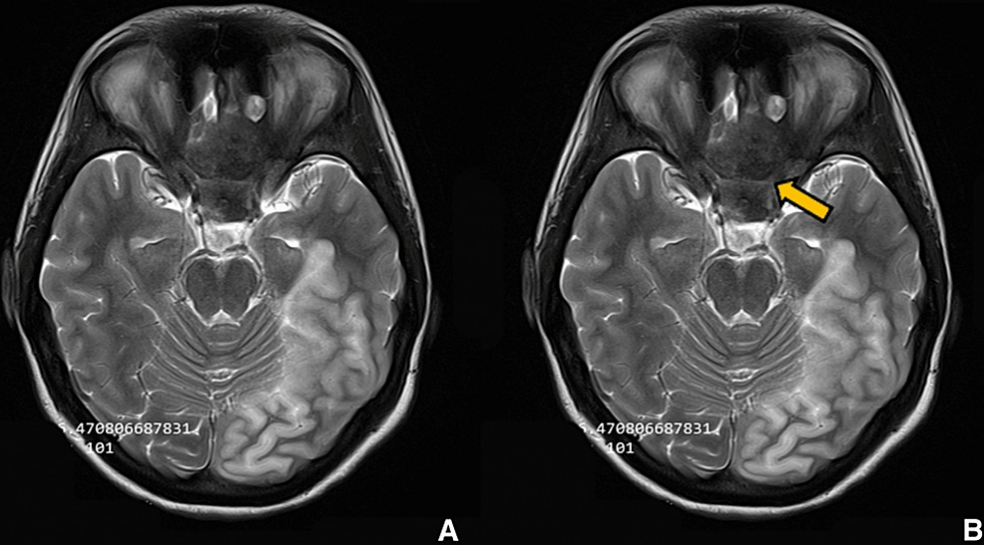
Preoperative imaging for patient 2. Plain T2-weighted axial image showing sellar, suprasellar lesion. (A) Without ROI and (B) with ROI.

**Figure 11:**
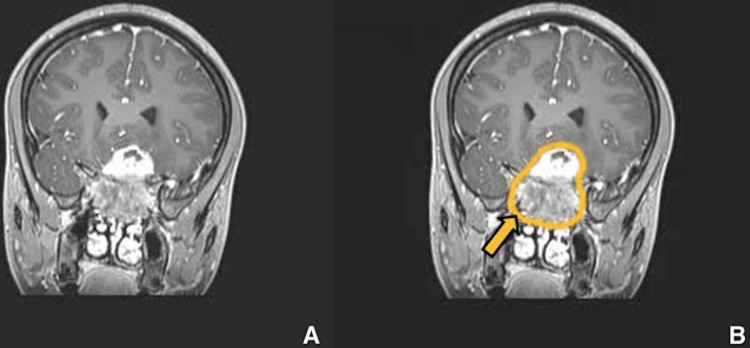
Preoperative imaging for patient 2. Contrast-enhanced T1-weighted coronal image showing sellar, suprasellar lesion with cavernous sinus invasion. (A) Without ROI and (B) with ROI.

**Figure 12:**
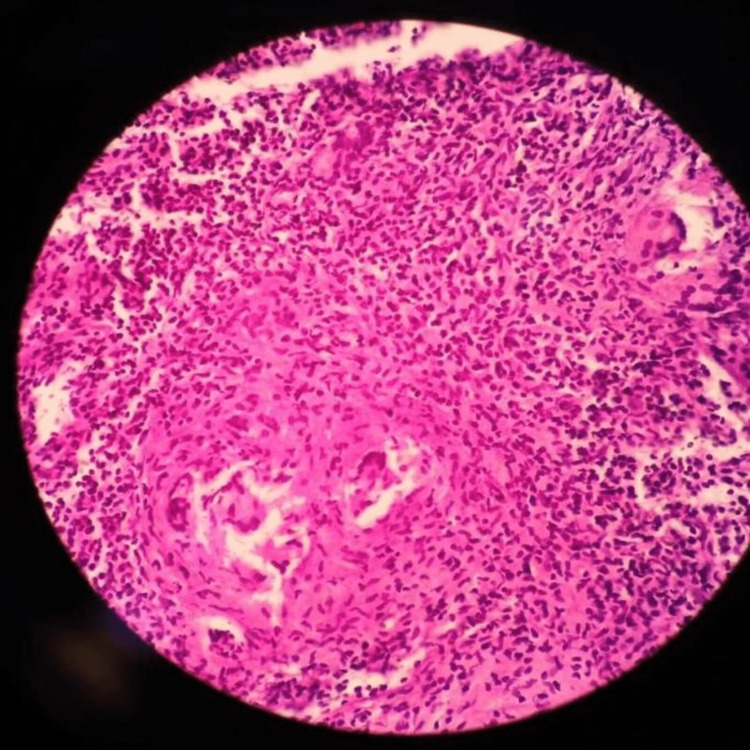
Pathology specimen image for patient 2. H&E stain showing acute on chronic inflammation with areas of necrosis.

**Figure 13:**
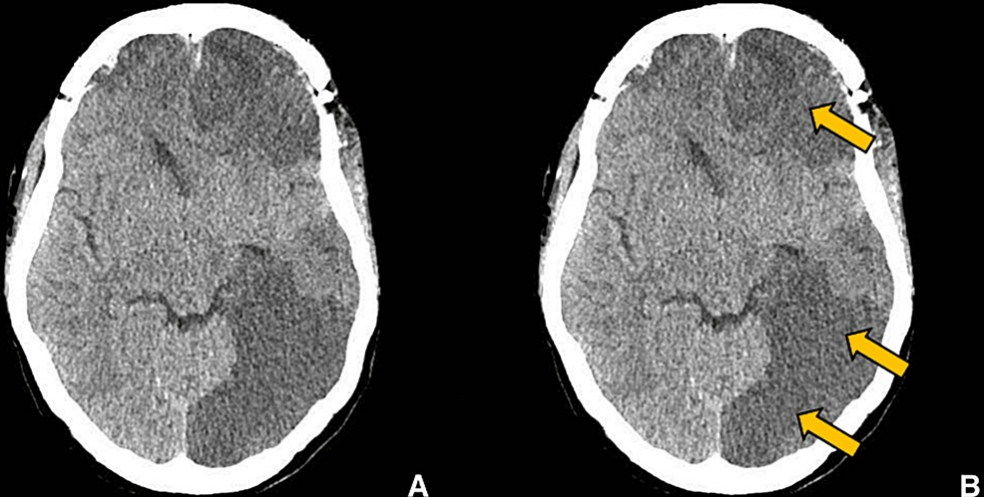
Postoperative imaging for patient 2. Non-contrast enhanced axial CT image showing multiple infarcts in bilateral middle cerebral artery and posterior cerebral artery territory. (A) Without ROI and (B) with ROI. The yellow arrows point to hypodense regions which show remnants of vessel infarction and decreased perfusion to the lesion following surgical debridement.

Initial MRI showed a heterogeneous enhancing mass involving sella, sphenoid sinus, and ethmoid sinus. The mass was extending intracranially through the cribriform plate, planum sphenoid, and the tuberculum sellae. The large suprasellar component measuring 3.6 x 4.7 x 8.5 cm was abutting the optic chiasm (Figures 9-11). The patient was emergently taken to the operating room due to rapid visual deterioration. A single-piece bifrontal craniotomy was performed, along with supraorbital rims which were removed bilaterally. An intraoperative basifrontal and suprasellar mass were identified. The tumor was hard, relatively avascular, and stuck to the optic chiasm, anterior communicating artery, and bilateral A2 arteries. The tumor was surgically decompressed after it was carefully dissected from the bilateral anterior cerebral artery and the optic chiasm. Subtotal resection of the tumor was achieved, and tissue affixed to the cerebral artery and chiasm was left behind. Histopathology revealed a large area of necrosis with entangled branched septate hyphae suggestive of *Aspergillus* species with acute inflammatory exudate with moderate to dense chronic inflammation (Figures 12A, 12B). The patient initially improved in sensorium, however, over the following 3 days postoperatively she became progressively drowsy. The patient’s serial CT scan revealed progressive infarcts involving bilateral frontal regions, the internal capsule, and the right middle cerebral artery territory (Figures 13A, 13B). Postoperatively the patient was started on liposomal Amphotericin-B at a very low dose (1 mg/kg/day) and voriconazole (400mg/day in divided doses). However, the patient’s sensorium continued to deteriorate over the next 3 days. On postoperative day 5, the patient became deeply comatosed with fixed and dilated pupils. She died on postoperative day 7 due to cerebral edema and infarcts. 

The patient’s immunologic function laboratory values and CBC are listed in Table 2. The patient had decreased CD3/CD4 counts at the time of diagnosis, which was consistent with her immunocompromised status. The CBC was significant for elevated ESR and eosinophils suggesting an acute immune response to fungal infection. No information was able to be obtained regarding other opportunistic infections. A further detailed medical history, including childhood history and vaccination status, was unable to be obtained for this patient.

**Table 2:** Laboratory studies from patient 2

Test	Results	Reference Range
Immune Deficiency Panel (Flow Cytometry)		
Absolute Lymphocyte Count	680/µL	990-3,150/µL
CD3 (Total T cells)	61.28%	59-83%
Absolute CD3	417/µL	677-2,383/µL
CD4 (Helper T cells)	27.51%	31-59%
Absolute CD4	187/µL	424-1,509/µL
Complete Blood Count		
Hemoglobin	13.0	11.5-14.5 g/dL
Total Leukocyte Count	6.3	4-10 x 10^3^/µL
RBC Count	4.34	4-5 x 10^6^/µL
ESR	50	mm/hr
Packed Cell Volume	39.3	37%-47%
Mean Corpuscular Volume	90.6	83-101 fL
Mean Corpuscular Hemoglobin	29.8	27-32 pg
MCHC	32.9	31.5-34.5 g/dL
RDW	12.5	11%-16%
Platelet	3.44	1.5-4 Lakhs/cumm
MPV	9.2	fl
Neutrophils	63	40%-80%
Lymphocytes	25	20%-40%
Monocytes	2	2%-10%
Eosinophils	10	1%-6%

## Discussion

Aspergilloma involving sphenoid sinus as well as clivus is relatively rare with only a few reported cases in the literature. Invasive aspergilloma is extremely uncommon in immunocompetent patients. *Aspergillus* sinus infections may develop in four distinct forms: allergic, noninvasive, invasive, and fulminant. Invasive aspergilloma can be a chronic indolent infection with slowly progressive disease as was present in patient 1, as discussed before, who was immunocompetent [4]. The invasive form of the disease may act in a similar manner as a malignancy in immunocompetent patients leading to a missed diagnosis. Invasive aspergilloma can also present as a rapidly progressive disease with an acute fulminant course as was present in patient 2 who was immunocompromised. The acute fulminant course of the disease often has up to a 70%-90% mortality rate in immunocompromised patients [5]. It is important to mention, though the disease course was different between these two patients due to their immune status, there were limited differences in the characteristics of their MR imaging, as discussed further below.

Epidemiology and prevention

It is known that cases of intracranial aspergillosis in immunocompetent patients without significant risk factors are very rare. A study examined the common characteristics of 182 patients presenting with intracranial aspergillosis. Among disease types, they identified 54.9% with skull base lesions, 23.6% with intraparenchymal disease, 13.2% with meningoencephalitis, and 8.2% with dural-based mass. Additionally, regarding the epidemiology of the disease, India, Pakistan, and Bangladesh had the highest prevalence in immunocompetent adult patients with 33% of the reported cases worldwide [6].

Due to the increased prevalence of intracerebral aspergilloma in immunosuppressed patients, certain recommendations are given to optimize the disease course in these patients. First, regarding treatment options, it is suggested that immunosuppressive therapy used in transplant patients should be reduced to increase the efficacy of antifungal therapy. Additionally, in severely neutropenic patients, adjunctive therapy with colony-stimulating factors and granulocyte transfusions may improve overall immunity and response to the disease. Lastly, adequate antifungal disease prophylaxis must be maintained in all immunocompromised patients with persistent fever [7].

The differential of mass lesions

The early clinical features of acute fulminant invasive fungal sinusitis include nasal obstruction, rhinorrhea, facial pain, headache, proptosis, and diplopia along with visual disturbances and cranial nerve involvement [3]. In our case series, patient 1 presented with headache and papilledema due to the presence of hydrocephalus. Patient 2 presented with acute headache, vomiting, and loss of vision in one eye, altered sensorium which was due to direct compression of the optic nerve and chiasm along with orbital edema. As mentioned, early invasive fungal sinusitis may present with benign features, or patients may even be completely asymptomatic; therefore, progression to symptoms of mass effect may take time. A short timeline of the mass effect of a sphenoid sinus aspergilloma begins with multiple nerve palsy involving the optic nerve and orbital apex. Additionally, the invasive fungal disease will eventually cause bony destruction of the pterygopalatine fossa and skull base, which can be well visualized on CT imaging. The walls of the sphenoid sinus hold many important neurovascular structures which also contribute to symptoms, including severe complications such as cavernous sinus thrombosis. The internal carotid artery, cavernous sinus, and cranial nerves can limit the ability to perform radical resection of invasive fungal disease [8].

The differential diagnosis of sinonasal mass with intracranial extension includes neoplasm, infection, and inflammation. Among neoplasms, an anterior skull base meningioma, trigeminal schwannoma, and large pituitary adenoma with multicompartmental suprasellar extent along with sphenoid sinus invasion can be confused with invasive fungal aspergilloma. These neoplasms commonly arise within the cavernous sinus and often cause necrotic disease and mass effect. Inflammatory pathologies like Wegener granulomatosis can also cause angioinvasive nasal disease and bony destruction which mimics skull-based aspergilloma, however, pulmonary, renal, and skin manifestations will also be present, along with strong c-ANCA positivity [3].

Disease management and prophylaxis

The standard of care for the treatment of invasive aspergillosis requires early and accurate diagnosis and subsequent initiation of antifungal therapy. The Infectious Disease Society of America (IDSA) recommends voriconazole as the initial therapy for invasive aspergillosis in most patients. In immunocompromised patients, combination therapy with echinocandins is suggested [7]. There is also a statistically significant difference in mortality of 16% in combination therapy (voriconazole and echinocandins) versus 27% in voriconazole monotherapy in patients with confirmed invasive pulmonary aspergillosis [9]. Voriconazole and other azole drugs are often preferred over amphotericin-B because of their better safety profile, as they carry a decreased risk of nephrotoxicity as well as infusion-related reaction. Liposomal formulations of amphotericin-B tend to be better tolerated, however, are very expensive compared to azole drugs [10]. Additionally, voriconazole can be used as an oral drug for long-term treatment, as the oral formulation carries a >90% bioavailability, the same as the intravenous formulation [11]. Throughout treatment, voriconazole efficacy is monitored by obtaining a serum trough concentration (therapeutic level between 1.0 and 5.5 mcg/mL) on patients four to seven days after the initiation of therapy. This is due to the high risk of voriconazole toxicity including visual changes, skin cancer, hepatotoxicity, and drug-drug interactions [7].

Surgery is indicated in almost all cases of intracranial aspergilloma with extensive necrotizing disease, as it not only histologically confirms the diagnosis, but also guides the degree of fungal involvement and allows for an early optimization of the adequate antifungal treatment regimen. Additionally, surgical debridement decreases the mass effect and relieves the pressure on the optic apparatus and other cranial nerves, thus providing more immediate symptomatic relief to patients. The angioinvasive nature of CNS aspergilloma also puts patients at risk for thrombosis or infarction; this ultimate progression to vessel invasion or intracerebral hemorrhage may be prevented by adequate surgical resection [6,10]. The different approaches of neurosurgical intervention include craniotomy with abscess resection, transnasal transsphenoidal approach, abscess drainage, or shunt placement. Sellar tumors are typically treated with a transnasal transsphenoidal approach, such as the cases reported in this paper. A study of 81 patients with intracranial aspergillosis found that neurosurgical intervention improved the disease course in 31% of patients [12]. 

Imaging modalities and other limitations

For patients experiencing significant neurologic symptoms imaging is often done as part of the initial workup. However, due to the limited differentiation of solid tumors based on imaging alone, an accurate diagnosis of cerebral aspergilloma is made with a combination of imaging, surgical histopathology, and clinical features. On MR imaging aspergilloma is hypointense on both T1WI and T2WI, whereas CT imaging tends to show a hyperdense lesion [10]. Additionally, MR imaging may show the presence of hypointense zones within areas of T2 hyperintense mass, which is specific to aspergillus infection. These hypointense zones may represent areas of active proliferation of aspergillus, as pathologic examination shows the increased iron concentration in these regions, the iron serving as an essential growth medium for fungal hyphae [13]. Compared to immunocompetent patients, immunocompromised patients with intracranial fungal mass can present as a ring-enhancing lesion, and while this is more suggestive of abscess formation, this difference in presentation may also complicate diagnosis, and raise concern for tumors in the immunocompromised. Abscesses are usually hyperintense on DWI, which can be differentiated from neoplasms which are usually hypointense or of variable intensity. A ring-enhancing abscess with an irregular wall and irregular projection into the cranial cavity with low ADC and no contrast enhancement likely suggests a fungal origin [3].

Therefore, DWI and ADC allow for excellent differentiation of fungal abscesses. Also, susceptibility-weighted MR sequences (SWMRS), must be mentioned due to gradient-echo sequences which have high sensitivity for observing products of byproducts of hemoglobin, iron, or calcifications. SWI imaging has shown to be helpful in differentiating pyogenic and fungal abscesses, where fungal abscesses lack a “dual-rim sign.” Though most fungal abscesses will show a hypointense ring on plain T2WI, this can be non-specific to a fungal etiology. In the future, SWI imaging may be useful for the diagnosis of these patients prior to surgical intervention [14].

Both CT and MRI are helpful in diagnosing the extent of aspergilloma and differentiating between invasive and non-invasive *Aspergillus* infection, but histological biopsy is ultimately needed to confirm the diagnosis. Through histopathology and culture, β-D-glucan levels are considered the most sensitive marker in patients with intracranial aspergillosis [3]. However, galactomannan, β-D-glucan test, and DNA detection of IA may have limited availability in medical centers worldwide [1]. PCR analysis of *Aspergillus* DNA also may be limited in use in certain hospital systems due to cost but has great potential due to its high sensitivity for the detection of the disease [5].

In regions of Western Europe, the rate of *A. fumigatus* resistance to triazoles (voriconazole, isavuconazole, and posaconazole) has increased to over 25%. Studies on antifungal resistance in countries outside of the United States and Europe remain limited [7]. However, antifungal resistance should always be considered in patients who do not respond to the treatment.

## Conclusions

In conclusion, sellar aspergilloma should be considered in the differential diagnosis of sellar and supra-sellar mass extending into the sphenoid sinus and clivus. Aspergilloma has a wide spectrum of clinical courses, including fulminant and rapid primarily in immunocompromised patients, and prolonged and indolent in immunocompetent patients. In our case series, we have highlighted differences in clinical features, radiological findings as well as clinical outcomes in immunocompromised and immunocompetent patients with invasive intracranial aspergilloma. The authors hope to highlight the need to further evaluate patients who specifically present with clinical symptoms of invasive fungal sinusitis, and who also show invasive and necrotic mass lesions on radiologic imaging. Multiple studies have proven that early diagnosis and adequate treatment with radical neurosurgical intervention combined with voriconazole antifungal therapy significantly reduces patient mortality. Thus, early diagnosis is imperative. Histologic analysis is still the mainstay of diagnosis of invasive aspergilloma; however, in the future noninvasive markers like β-D-glucan levels, PCR analysis, or SWMRS imaging may help us in making an early accurate diagnosis.
